# Urban Environmental Threat Moderates the Relationship Between Depression and Insulin Resistance Among Latinxs With Type 2 Diabetes

**DOI:** 10.1002/smi.3504

**Published:** 2024-11-23

**Authors:** Kevin A. Matlock, Maggie R. Albright‐Pierce, Angela Bermúdez‐Millán, Rafael Pérez‐Escamilla, Sofia Segura‐Pérez, Julie Wagner

**Affiliations:** ^1^ Department of Psychiatry University of Oxford Oxford UK; ^2^ Worcester College University of Oxford Oxford UK; ^3^ Department of Psychology Rutgers University Piscataway New Jersey USA; ^4^ Department of Public Health Sciences University of Connecticut Farmington Connecticut USA; ^5^ Yale School of Public Health Yale University New Haven Connecticut USA; ^6^ Nutrition Unit Hispanic Health Council Hartford Connecticut USA; ^7^ University of Connecticut Health Center University of Connecticut Farmington Connecticut USA

**Keywords:** depression, insulin resistance, Latinx, type 2 diabetes, urban environmental threat

## Abstract

As the largest minoritised ethnic group in the United States, Latinxs face a greater risk for type 2 diabetes and depression. The aim of the present study was to explore whether the relationship between depressive symptoms and insulin resistance among Latinxs with type 2 diabetes was moderated by toxic stressors arising from urban environmental threat (i.e., uncomfortable or unsafe aspects of city life). A community sample of Latinx adults with type 2 diabetes (*n* = 121) was recruited from Hartford, Connecticut. Participants self‐reported depressive symptoms and exposure to urban environmental threat using items from the Patient Health Questionnaire and Urban Hassles Index, respectively. Insulin and glucose levels assessed via fasting blood draw were used to calculate insulin resistance using the HOMA‐IR formula. After controlling for demographic, financial and health‐related factors, results from a regression analysis revealed a significant interaction between depressive symptoms and urban environmental threat; more severe symptoms of depression predicted greater insulin resistance, but only amongst those with frequent exposure to urban environmental threats. Findings from the current study suggest that improving urban living conditions may offer an alternate avenue for attenuating the deleterious impacts of depression on type 2 diabetes progression in Latinxs.

## 1 Introduction

Latinx—a gender‐inclusive term for those who identify as Latina/Latino or Hispanic—is the largest minoritised ethnic group in the United States, comprising 19% of the total population (Funk and Lopez 2022). Diabetes risk for this group remains disproportionately high. Estimates vary among subethnicities (Schneiderman et al. 2014), but overall prevalence for type 2 diabetes in Latinxs (12%) is nearly twice that of non‐Hispanic White persons (7%; CDC 2022). As such, developing models to better understand the progression of type 2 diabetes in this population is vital for improving care.

### 1.1 Role of Depression

Depression risk is higher in both the type 2 diabetes and Latinx populations, for whom the odds of developing depressive symptoms are 90% higher (Farooqi et al. 2022) and 80% higher (Mikolajczyk et al. 2007) than the general population, respectively. Depression is also associated with *insulin resistance*, an underlying, defining characteristic of type 2 diabetes in which the body loses sensitivity to insulin, leading to elevated blood glucose (Fernandes et al. 2022; Kan et al. 2013). Mechanisms underlying this association are not fully understood, but evidence points towards a potential bi‐directional relationship where (a) insulin resistance acts as a route to depression by interfering with how stress is regulated (Lyra e Silva et al. 2019) and (b) depression acts to increase insulin resistance via mediating health‐related factors like waist circumference, which are also linked to stress (Pearson et al. 2010). Thus, symptoms of depression follow not only as a consequence of type 2 diabetes but may contribute to its progression as well by increasing resistance to insulin.

### 1.2 Role of Environment

Latinxs in urban communities often report neighbourhood crime and violence as significant sources of stress (Bermúdez‐Millán et al. 2011; Mendenhall and Jacobs 2012)—toxic stressors which are implicated in the aetiology of type 2 diabetes (Walsan et al. 2018) and depression (Barnett et al. 2018). Residents in densely populated areas are also more likely to encounter noxious environmental stressors like noise and pollution (Lederbogen et al. 2011) and, in a similar fashion, this increased stress can further exacerbate symptoms of diabetes (Lloyd, Smith, and Weinger 2005) and depression (Peen et al. 2010). Various terms have been used to describe the deleterious effects of city life; here the phenomenon is termed *urban environmental threat*.

### 1.3 Aim

Early systematic reviews supported an association between diabetes and depression, but not all studies reported significant findings and effect sizes varied (Yu et al. 2015), while a recent meta‐analysis of longitudinal studies points towards a bidirectional relationship between depression and glycaemia (Beran et al. 2022). There is also a paucity of research on the impacts of urban living (Walsan et al. 2018), and its influence on this relationship has not been investigated. Therefore, to explore these factors, a secondary analysis tested whether urban environmental threat moderated the relationship between depression and insulin resistance in Latinx adults with type 2 diabetes.

## 2 Method

### 2.1 Participants

Data were sourced from the Community health educators Assisting Latinos Manage Stress and Diabetes (CALMS‐D) study (Wagner et al. 2015). In CALMS‐D, outpatients were recruited from an urban community clinic in Hartford, Connecticut, for a larger study on enhanced care. Participants were included in the study if they self‐identified as Latino/Latina or Hispanic and were ambulatory adults diagnosed with type 2 diabetes for at least 6 months. Participants were excluded if they screened positive for bipolar, substance, or thought disorders; serious medical conditions precluding participation; or recent suicide attempt or psychiatric hospitalisation.

### 2.2 Procedure

In‐home interviews in CALMS‐D were conducted between 2012 and 2014 by trained community health workers in participants' language of choice (Spanish or English). Survey responses were recorded using self‐report and the Remote Electronic Data Capture (REDCap) tool (Harris et al. 2009). Fasting blood samples were drawn by a phlebotomist during home visits and transported to a laboratory for processing. Participants were paid 10 USD for each interview and for each blood draw. For details about translation, recruitment and laboratory procedures, see Wagner et al. (2015).

### 2.3 Measures

#### 2.3.1 Depressive Symptoms

The 8‐item Patient Health Questionnaire (PHQ‐8; Kroenke et al. 2009) assessed how often participants experienced symptoms of depression in the last 2 weeks. The PHQ‐8 is comprised of items like ‘feeling down, depressed, or hopeless’ and ‘feeling tired or having little energy’ rated on a scale from 0 (*not at all*) to 3 (*nearly every day*). Scale reliability was acceptable (*α*_*c*_ = 0.8).

#### 2.3.2 Urban Environmental Threat

The Urban Hassles Index (UHI; Bennett and Miller 2006) measures a range of stressors specific to adolescents in urban settings. The 3‐item UHI Social Disorganisation Subscale was used to capture urban environmental threat by assessing how often in the past year, on a scale from 0 (*never*) to 3 (*very often*), participants were bothered by ‘noisy neighbours, loud cars, dirty bus stops, or abandoned buildings’, felt ‘nervous because of living in or near an unsafe area’, or were ‘afraid of strangers’ because of problems with ‘drugs, violence, or crime’. To adapt this measure for adults, a fourth UHI item was included that assessed how often participants had their ‘belongings stolen or … residence or car broken into’. The addition of this item improved reliability, increasing scale reliability (*α*_*c*_ = 0.6) to reach the lowest threshold of acceptability (*α*_*c*_ ≥ 0.6; El Hajjar 2018).

#### 2.3.3 Insulin Resistance

Laboratory assessments for fasting serum insulin and plasma glucose were used to calculate insulin resistance via the Homoeostatic Model Assessment of Insulin Resistance (HOMA‐IR) formula (Matthews et al. 1985). Higher HOMA‐IR values reflect greater resistance to insulin and signal more advanced progression of type 2 diabetes.

#### 2.3.4 Covariates

Gender, age, employment and monthly household income in 500 USD increments were assessed via self‐report. Financial strain was rated on a scale from 1 (*have enough and can save*) to 5 (*don't have enough and have great difficulties*). Waist‐to‐hip ratio was calculated by dividing waist circumference by hip height, with both measures taken in centimetres by trained staff. Serum total and high‐density lipoprotein (HDL) cholesterol in mg/dL were obtained by laboratory assessment. To avoid redundancy, only HDL cholesterol was retained due to a lack of association between total cholesterol and other variables. The demographic, financial and health‐related factors above were considered as confounders because they (a) share important associations with diabetes and depression (Asghar et al. 2007; Brown et al. 1994; Chaufan and Weitz 2009; Sandhu, Koley, and Sandhu 2008) and (b) were necessary to differentiate between general socioeconomic disadvantage (Campbell and Campbell 2007) and the specific uncomfortable and unsafe elements that constitute urban environmental threat.

### 2.4 Analysis

Continuous variables retained their original coding, with more positive values reflecting older age, greater financial strain, higher HDL (‘good’) cholesterol, greater abdominal adiposity, more severe depression, more frequent experiences with urban environmental threat and greater insulin resistance. Two categorical variables were dichotomised for analysis due to low endorsement of other response options: Employment status was recoded as either employed or unemployed, and monthly household income was dichotomised as either above or at/below the federal poverty line for a single‐person household (1000 USD; Office of the Assistant Secretary for Planning and Evaluation 2015).

Analyses were conducted in *R* version 4.2.2 (R Core Team 2022) using cross‐sectional baseline data from CALMS‐D and pairwise deletion of missing value. Bivariate relationships used Pearson's correlation and *psyc* package version 2.2.9 (Revelle 2022). Direct effects were analysed using multiple linear regression models with depression and urban environmental threat entered as predictors; insulin resistance as the outcome; and age, gender, employment status, income, financial strain, waist‐to‐hip ratio and/or HDL cholesterol as covariates. Moderation was tested by adding a depression‐by‐environmental‐threat interaction term to the final model. Prior to analysis, number of subjects per variable for the final model was confirmed to meet guidelines for adequately estimating coefficients and probabilities (Austin and Steyerberg 2015). Standardised regression coefficients and simple slopes tests for interactions were obtained using *reghelper* package version 1.1.1 (Hughes and Beiner 2022). Differences in model fit were significance‐tested using ANOVA.

## 3 Results

### 3.1 Sample

The sample was comprised of 121 Latinx adults with type 2 diabetes. Participants were predominantly women between 21 and 86 years old. Most had a monthly income at/below the federal poverty threshold, and half reported financial strain. A sizeable portion of the sample (29%) exceeded the threshold for clinical depression (PHQ ≥ 10; Kroenke et al. 2009), half experienced one or more incidents of urban environmental threat in the last year, and a majority (77%) had insulin resistance levels in the top quintile for the general population (HOMA‐IR > 4.1; Ruijgrok et al. 2018). Sample characteristics and correlations are listed in Table 1.

**TABLE 1:** Sample characteristics and bivariate correlations.

Variable	*M* (*SD*) or *Mode* (%)	1	2	3	4	5	6	7	8	9
1. Insulin resistance (HOMA‐IR)	12.95 (13.70)									
2. Urban environmental threat (UHI)	1.48 (2.00)	0.16								
3. Depression (PHQ‐8)	6.22 (5.46)	0.18	0.47***							
4. Age (years)	60.68 (11.63)	−0.12	0.08	−0.18						
5. Waist‐to‐hip ratio	1.50 (0.83)	0.14	−0.01	−0.01	−0.01					
6. Cholesterol (HDL)	0.98 (0.12)	−0.23*	0.06	−0.03	0.20*	−0.30***				
7. Gender^a^	Female (74%)	0.22*	0.04	−0.07	−0.06	0.21*	−0.24**			
8. Employment^a^	Unemployed (90%)	−0.21*	−0.06	0.07	0.22*	−0.08	0.15	−0.06		
9. Monthly income^a^	$1000 or less (67%)	−0.24**	−0.03	0.02	0.17	0.05	0.19*	−0.10	0.24**	
10. Financial strain	Just enough (40%)	0.02	0.15	0.14	0.07	0.24**	0.07	0.08	0.07	−0.04

*Note:* Descriptives given as mean (standard deviation) for continuous variables and mode (% of sample) for categorical variables.

Abbreviations: HDL, high‐density lipoprotein; HOMA‐IR; homoeostatic model assessment of insulin resistance; PHQ‐8, patient health questionnaire 8‐item; UHI, urban hassles index.

^a^ Dichotomous variable; reference groups are female (vs. male), unemployed (vs. employed) and monthly income at/below 1000 USD (vs. above).

*p < 0.05, **p < 0.01, ***p < 0.001.

### 3.2 Insulin Resistance

#### 3.2.1 Model Comparisons for Main Effects

An initial regression model was constructed with depression and urban environmental threat entered as predictors, and age and gender entered as demographic covariates. This model significantly predicted degree of insulin resistance, *R* ^2^ = 0.13, *F* (4,108) = 3.3, *p* = 0.02. To control for confounding due to financial and health‐related factors, a second model added three more covariates: employment, income and HDL cholesterol. Their inclusion boosted variance explained by more than 50% relative to the initial model, ∆*R* ^*2*^ = 0.07, *F* (3,105) = 3.2, *p* = 0.03. A third model added financial strain and waist‐to‐hip ratio to the second model. Model comparisons were done in stepwise fashion to preserve model parsimony, and covariates in the third model were added last as they were uncorrelated with the outcome. The increase in variance explained by adding these two covariates was negligible compared to the previous model, ∆*R* ^2^ < 0.01, *F* (2,103) < 1.0, *p* = 0.73. Therefore, to balance parsimony with explanatory power, interpretations for main effects and moderation tests were based on the second model, *R* ^2^ = 0.18, *F* (7,106) = 3.4, *p* = 0.003.

#### 3.2.2 Moderation of Depression by Urban Environmental Threat

Moderation was tested by adding a depression‐by‐urban‐environmental‐threat interaction term to a regression model with gender, age, employment, income and HDL cholesterol entered as covariates. As a set, these variables explained nearly one quarter of insulin resistance variability—a significant improvement over the non‐interaction model, ∆*R* ^2^ = 0.03, *F* (1,105) = 4.6, *p* = 0.04. Results for the final moderation model are listed in Table 2.

**TABLE 2:** Regression model predicting insulin resistance (HOMA‐IR).

Variable	*β*	*t*	*p*
Model			
*R* ^2^ = 0.22, *F* (8,105) = 3.6			< 0.001***
Covariates			
Age (years)	< 0.01	0.1	0.926
Cholesterol (HDL)	−0.13	1.4	0.169
Gender^a^	0.18	2.0	0.049*
Employment^a^	−0.18	2.0	0.052
Monthly income^a^	−0.15	1.6	0.109
Predictors			
Urban environmental threat (UHI)	−0.06	0.5	0.629
Depression (PHQ‐8)	0.17	1.6	0.119
Interaction (UHI × PHQ‐8)	0.22	2.1	0.035*

Abbreviations: HDL, high‐density lipoprotein; HOMA‐IR; homoeostatic model assessment of insulin resistance; PHQ‐8, patient health questionnaire 8‐item; UHI, urban hassles index.

^a^ Dichotomous variable; reference groups are female (vs. male), unemployed (vs. employed) and monthly income at/below 1000 USD (vs. above).

*p < 0.05, **p < 0.01, ***p < 0.001.

As independent main effects, neither depression nor urban environmental threat significantly predicted insulin resistance. Likewise, none of the covariates were significant predictors, apart from gender where men displayed greater resistance to insulin than women. The absence of main effects was qualified, however, by a significant interaction between depression and urban environmental threat. This interaction was decomposed using simple slopes tests. Results demonstrated that, when predicting insulin resistance, slopes for depression were small and non‐significant when urban environmental threat was low (values 1.0 *SD* below the mean), *β* = −0.13, *p* = 0.74, or average (values at the mean), *β* = 0.13, *p* = 0.12. However, when frequency of urban environmental threat was high (values 1.0 *SD* above the mean), the slope for depression was moderate and significant, *β* = 0.39, *p* = 0.009, with more severe symptoms of depression predicting greater insulin resistance.

## 4 Discussion

The present study investigated depression and urban environmental threat (i.e., uncomfortable and unsafe aspects of city life) as predictors of insulin resistance in Latinx adults with type 2 diabetes. After controlling for key demographic, financial and health‐related factors, neither depressive symptoms nor incidents of urban environmental threat shared significant associations with insulin resistance when evaluated as independent effects. While it is possible these effects may have been detectable with a larger sample, results may also be due to a qualifying moderation effect. Considered together, results supported moderation whereby more severe symptoms of depression predicted greater resistance to insulin, but only amongst those with frequent exposure to urban environmental threats. If replicated in larger samples, these findings may suggest experiencing depressive symptoms and residing in a stressful urban setting combine to accelerate the progression of type 2 diabetes.

To our knowledge, this study is the first to assess this type of moderation for insulin resistance. Previous studies have reported that impoverished living conditions may contribute to the aetiology of type 2 diabetes (Chaufan and Weitz 2009), and depressive symptoms may contribute to insulin resistance (Kan et al. 2013). However, few studies have examined diabetes and the deleterious effects of urban life (Walsan et al. 2018), and none have explored these vulnerabilities together in the context of depression. As such, the current study is novel not only in assessing depression and urban environmental threat within the same model, but also in finding evidence to support moderation—a discovery which may explain heterogeneous results in previous depression studies (Kan et al. 2013; Yu et al. 2015).

Still, underlying mechanisms remain unclear. While causal processes cannot be determined due to the cross‐sectional nature of this study, it is possible to speculate about why moderation may have occurred. One hypothesis is that moderation may be attributable to disruptions in emotion and stress regulation. Previous studies report that difficulties with emotion regulation are linked to difficulties in managing interpersonal stress which, in turn, are linked to increases in depressive symptoms (Moriya and Takahashi 2013). Similarly, in the present study, participants with more severe depression may also have had greater difficulty managing stress related to urban environmental threats (e.g., noise, theft, violence). Thus, depressogenic vulnerability to stress and exposure to a stressful urban setting may have acted in concert to worsen type 2 diabetes progression (Lloyd, Smith, and Weinger 2005; Walsan et al. 2018). Indeed, the stressful nature of life in an urban setting is reflected physiologically by the elevated amygdala activity of its residents (Lederbogen et al. 2011)—activity which is compounded by inflammatory biomarkers associated with depression (Leonard and Wegener 2020).

An alternate hypothesis could be that urban environments exacerbate depressive symptoms directly, which then leads to greater insulin resistance. However, this depression‐pathway explanation was tested *post hoc* using data from the present study and, when assessed with a similar covariate‐adjusted model, no evidence was found to support a mediation effect for depression (see Supporting Information S1: Appendix 1). Another plausible explanation is that environment restricts health‐related behaviour. Living in unpleasant or dangerous urban areas may leave residents feeling reluctant to venture outside, and for those who experience the motivational difficulties that typically characterise depression (Smith 2013), this avoidance pressure may create serious barriers for engaging in diabetes self‐care behaviours that protect against insulin resistance. For instance, remaining indoors may lessen physical activity, a lifestyle factor which has been shown to prevent, reduce and in some cases reverse insulin resistance (Whillier 2020). To account for this possibility, self‐report data for exercise were examined in the current study. A near‐zero, non‐significant correlation was found between days exercised per week and insulin resistance, and adding exercise as a predictor had no impact on the moderation model, suggesting that physical activity could not account for this study's findings.

Finally, urban environments have the potential to negatively impact health through diet. Low‐income urban areas are commonly afflicted by ‘food deserts’ where healthy dietary options require substantial travel time to reach (Bermúdez‐Millán et al. 2011; Ghosh‐Dastidar et al. 2014), and restricted access to healthy foods is associated with insulin resistance (Misra et al. 2008). Moreover, depression is thought to increase insulin resistance through mediating health‐related factors like waist circumference (Pearson et al. 2010). Nevertheless, in the present study, the interaction remained significant despite controlling for health‐related factors such as HDL cholesterol and wait‐to‐hip ratio.

### 4.1 Contributions and Implications

In addition to being a novel finding, the discovery that depression and urban environmental threat were co‐occurring risk factors for insulin resistance has important implications for understanding long‐term progression of type 2 diabetes. While downstream indicators of metabolic health, for instance glycaemic control (i.e., HbA1c), are easily assayed via finger‐prick, they offer less discriminatory power as they are subject to greater confounding from factors like treatment type and medication adherence (Sharma and Fleming 2012). In contrast, though more difficult to assess because it requires a fasting blood draw, insulin resistance tends to be a more reliable indicator of long‐term metabolic health because it is a relatively upstream feature of diabetes progression (Sharma and Fleming 2012).

Furthermore, that no significant correlations were found between urban environmental threat and socioeconomic indicators like income, employment, or financial strain may suggest elevations in insulin resistance arising from urban environment threat are distinct from those attributable to overall socioeconomic status. It is possible, however, that these results occurred due to ceiling effects. More than 95% of the sample in this study had yearly incomes below 24,000 USD, whereas an earlier study with a more socioeconomically diverse sample reported that yearly incomes of 75,000 USD were associated with a lower diabetes prevalence than incomes of 20,000 USD (Schneiderman et al. 2014). Nevertheless, taken together, the findings in the present study underscore previous calls for a biopsychosocial approach to understanding the natural life course of type 2 diabetes (Kalra, Baruah, and Sahay 2018).

### 4.2 Limitations and Recommendations

Models in the current study were tested using data from a small sample recruited from one clinic in 2015, which may limit confidence in the results and their generalisability to other groups and time periods. The cross‐sectional analysis also did not allow for definitive claims about causality or directionality. Analyses of longitudinal data from more demographically and socioeconomically diverse samples are needed to confirm whether depression and urban environmental threat precede or follow changes in insulin resistance in the wider Latinx population. Additionally, few measures exist for urban environmental threat. Despite choosing the most suitable option available, this study adapted an instrument validated on a sample of predominantly Black adolescents, which may explain why scale reliability was at the lower threshold of acceptability. The subscale was also constrained to four items, which may have further reduced reliability and sensitivity (El Hajjar 2018). Future validation studies incorporating larger, more diverse samples and new environmental threats—especially those important for the Latinx population, such as discrimination (Matlock, Pérez‐Escamilla, and Wagner 2024)—are needed, as are studies exploring and testing potential mechanisms underlying the moderation effect.

### 4.3 Conclusion

Latinxs, the largest minoritised ethnic group in the United States, have higher rates of type 2 diabetes and depression. Findings from the present study shed light on how these factors are influenced by living conditions, and serve to demonstrate that, amongst Latinx adults with type 2 diabetes, depressive symptoms are associated with greater insulin resistance, but only when urban environmental threat is high. This moderation effect has important implications for intervention studies seeking to resolve health inequities and improve care for at‐risk groups.

## Ethics Statement

CALMS‐D was conducted in accordance with the Helsinki Declaration and received ethics approval from Institutional Review Boards at UCONN Health, Yale University, Hartford Hospital and the Hispanic Health Council.

## Conflicts of Interest

The authors declare no conflicts of interest.

## Supporting information

Supporting Information S1

## Data Availability

This secondary analysis relied upon a pre‐existing dataset. For enquiries and requests concerning access to the original data that supported this study, please contact the corresponding author for CALMS‐D (Wagner et al. 2015).
