# Supplementary material for: Urban Environmental Threat Moderates the Relationship Between Depression and Insulin Resistance Among Latinxs With Type 2 Diabetes
Source: Stress Health. 2024 Nov 23;40(6):e3504. doi: 10.1002/smi.3504 (PMC11636443; doi:10.1002/smi.3504)
Supplement: Supplementary file 1 — Supporting Information S1 [file SMI-40-e3504-s001.docx]

| **Appendix 1. Figure Depicting Model Testing Depression as a Mediator for Urban Environmental Threat and Insulin Resistance** |
| --- |
| β = .07  β = .58***  Urban Environmental Threat  (UHI)  Insulin Resistance  (HOMA-IR)  β = .18  Depressive Symptoms  (PHQ-8) |
| *Abbreviations:* HDL, high-density lipoprotein; HOMA-IR; Homeostatic Model Assessment of Insulin Resistance; PHQ, Patient Health Questionnaire; UHI, Urban Hassles Index.  *Note:* Following bootstrapping with 1,000 repetitions, significance testing for a regression model with gender, age, employment, income, and HDL cholesterol entered as covariates did not support a mediation pathway for depression, β = .04, *p* = .55.  *** *p* < .001. |
